# Gestational Trophoblastic Neoplasia Rate and Its Related Factors in Women With a Partial Hydatidiform Mole at Tudu Hospital, Vietnam

**DOI:** 10.7759/cureus.67495

**Published:** 2024-08-22

**Authors:** Tuan M Vo, Tuyet T Hoang, Hoang M Tran, Kimberley Nyamakope

**Affiliations:** 1 Obstetrics and Gynaecology (OB-GYN), University of Medicine and Pharmacy at HCMC (Ho Chi Minh City), Ho Chi Minh, VNM; 2 Diagnostic Radiology, University of Medicine and Pharmacy at HCMC (Ho Chi Minh City), Ho Chi Minh City, VNM; 3 Foundation School, Somerset NHS Foundation Trust, Somerset, GBR

**Keywords:** female patient, partial hydatidiform mole, gestational trophoblastic neoplasia, choriocarcinoma, chemoprophylaxis

## Abstract

Background

Minimal studies have been carried out on a partial hydatidiform mole (PHM) in Vietnam, so the treatment outcomes for patients with PHM are unknown. This study aimed to determine the occurrence rate of gestational trophoblastic neoplasia (GTN) and its related factors in women with PHM at Tu Du Hospital, Vietnam.

Materials and methods

This retrospective cohort study included 370 women with PHM diagnosed through a histopathological assessment following termination of pregnancy at Tu Du Hospital from January 2020 to December 2021. Survival analysis was used for GTN cumulative rate estimation and the Cox regression model for determining GTN-related factors.

Results

After a 1-year follow-up, 21 patients were found to have GTN, exhibiting a rate of 5.7% (95% confidence interval (CI): 3.5 - 8.4). GTN occurred 4.67±2.23 weeks following curettage with peaks at weeks 3-6. No cases of GTN were recorded eight weeks following termination by curettage. After multivariate analysis, the GTN rate was higher in patients with a history of miscarriage/termination (hazard ratio (HR)=2.84; 95% CI: 1.05-7.69).

Conclusion

The rate of GTN in PHM patients was 5.7%. Patients who had a history of miscarriage or termination were 2.84 times more likely to develop GTN than patients who did not.

## Introduction

A hydatidiform mole (HM) is one of the most frequent manifestations of gestational trophoblastic disease (GTD) due to an abnormal proliferation of placental tissue. In a complete hydatidiform mole (CHM), there is no fetal tissue present, whereas a partial hydatidiform mole (PHM) refers to instances where fetal tissue coexists with degenerated placental tissue. The natural progression and prognosis of these two types of HM differ significantly due to their pathophysiological mechanisms [1-3]. About 80% of cases of HM will revert to normal following termination by curettage. However, there is still a risk of malignancy with invasive and metastatic features, including invasive mole (15%) and choriocarcinoma (5%) [4,5]. The progression to an invasive mole or choriocarcinoma depends on the classification of HM, where malignancy is more likely with CHM than with PHM. Nevertheless, the possibility of malignancy through PHM cannot be ignored [6].

The rate of progression to GTN in patients with PHM diagnosed through post-termination histopathological assessment is approximately 4-6% [7,8]. Chemoprophylaxis and preventive hysterectomy may reduce the risk of developing GTN, but efficacy is controversial and cannot replace rigorous post-termination management measures. Understanding the rate of GTN and its related factors in women with PHM helps provide better counseling, preventive treatment, and follow-up [4,5].

Vietnam has a high prevalence of HM. Tu Du Hospital is the major center for its management, treatment, and follow-up in the southern provinces of Vietnam. Annual statistics compiled by the Gynecologic Oncology Department of Tu Du Hospital disclose that the hospital provides treatment and follow-up services to 800-1000 HM cases a year. For example, from January 2019 to December 2022, the hospital received 815 cases of PHM alone [9]. Minimal studies have been carried out on PHM in Vietnam, so the treatment outcomes for patients with PHM are unknown. This study aims to determine the GTN rate and its related factors in women with PHM at Tu Du Hospital.

## Materials and methods

Study design

This study used a retrospective cohort design.

Study setting.

The study was conducted at the Department of Gynecologic Oncology, Tu Du Hospital, Ho Chi Minh City, Vietnam.

Study participants

Medical records of patients diagnosed and admitted to Tu Du Hospital with PHM from January 2020 to December 2021 were collected. The inclusion criteria were as follows: 1) available histopathologic assessment after termination by curettage, which is confirmed PHM, 2) no evidence of on-site invasion or metastasis, and 3) monitoring as per Tu Du Hospital’s guidelines for 1 year or until full recovery (at least 6 months since β-hCG negative for low-risk PHM or 12 months for high-risk HM).

The exclusion criteria included: 1) patients who became pregnant during the follow-up period, 2) patients who received a hysterectomy due to other causes rather than undergoing ongoing treatment for GTD, or 3) being lost to follow-up in the treatment process or having missing information in their medical records.

Sample size and sampling procedure

The total sampling period was from January 2020 to December 2021. Data collected from 370 inpatient and outpatient medical records contained basic epidemiologic information, PHM features, post-termination treatment methods, and time of GTN occurrence. At Tu Du Hospital, post-termination PHM patients are monitored on beta-human chorionic gonadotropin (β-hCG) bi-weekly until three consecutive negative tests, then monthly for 6 consecutive months, followed by twice a month for the next 6 months and once every 3 months for the next 12 months.

Evaluation parameters

We used the FIGO (Fédération Internationale de Gynécologie et d’Obstétrique) 2000 criteria [10] to diagnose GTN post-PHM including: 1) a β-hCG increase of greater than 10% across three consecutive tests in two weeks (days 1, 7, and 14), 2) β-hCG plateau across four consecutive tests in three weeks (days 1, 7, 14, and 21), and 3) choriocarcinoma by histopathologic diagnosis. GTN occurrence was measured in weeks, as the time between curettage termination and a confirmed diagnosis.

Statistical analysis

Data were entered and analyzed with STATA 14 (StataCorp LLC, Lakeway Drive College Station, TX, USA). A survival table was used to estimate the cumulative GTN rate. Univariable and multivariable Cox regression models were used to determine the correlation between risk factors and GTN incidence. Variables applied to the multivariable Cox regression model included those with a p-value of <0.25 in univariable analysis and some with a known GTN relation.

Ethical considerations

This study was conducted in accordance with the Declaration of Helsinki. The Institutional Review Board of Tu Du Hospital granted ethical approval for the conduct of this study (No: 2042/BVTD-HĐĐĐ).

## Results

Among 370 patients enrolled in the study from January 2020 to December 2021, 21 patients developed GTN. The epidemiologic and clinical features of the disease are presented in Table 1. Patients’ ages ranged between 17 and 56 years; mean age was 32.9±7.7 years. The proportion of patients who have never given birth was high, at 145/370 (39.9). Of the patients, 165/370 (44.6%) had a previous history of miscarriage/termination. The most common clinical symptoms were vaginal bleeding 122/370 (33.0%). Most patients had blood β-hCG levels >100,000 mUI/mL (214/370 (57.8%)). Most patients with PHM were classified as low risk according to the WHO 1983 classification (368/370 (85.4%)) [11]. The initial intergroup analysis demonstrated a non-statistically significant difference in GTN incidence between the following groups: patients >40 years of age, β-hCG concentration of greater than 100,000 mUI/mL at the time of diagnosis, and a low-risk classification according to the WHO 1983 classification.

**Table 1:** Epidemiologic and clinical features of partial hydatidiform mole patients (n=370) GTN: gestational trophoblastic neoplasia; β-hCG: beta-human chorionic gonadotropin; *: Logrank test

Features	Total (%) (n=370)	Remission (%) (n=249)	GTN (%) (n=21)	p-value*
Age (Years)				
<30	133 (35.9%)	130 (97.7%)	3 (2.3%)	0.21
30-40	171 (46.3%)	160 (93.6%)	11 (6.4%)	
>40	66 (17.8%)	59 (89.4%)	7 (10,6%)	
Number of births				
Not yet	145 (39.2%)	140 (96.6%)	5 (3.4%)	0.49
1 time	127 (34.3%)	119 (93.7%)	8 (6.3%)	
≥2 times	98 (26.5%)	90 (91.8%)	8 (8.2%)	
History of miscarriage or abortion				
No	205 (55.4%)	199 (97.1%)	6 (2.9%)	0.04
Yes	165 (44.6%)	150 (90.9%)	15 (9.1%)	
Vaginal bleeding				
No	248 (67.0%)	237 (95.6%)	11 (4.4%)	0.44
Yes	122 (33.0%)	112 (91.8%)	10 (8.2%)	
β-hCG at the time of diagnosis (mUI/mL)				
<100.000	156 (42.2%)	152 (97.4%)	4 (2.6%)	0.11
≥100.000	214 (57.8%)	197 (92.1%)	17 (7.9%)	
Risk by WHO 1983				
Low	316 (85.4%)	303 (95.9%)	13 (4.1%)	0.12
High	54 (14.6%)	46 (85.2%)	8 (14.8%)	

**Figure 1:**
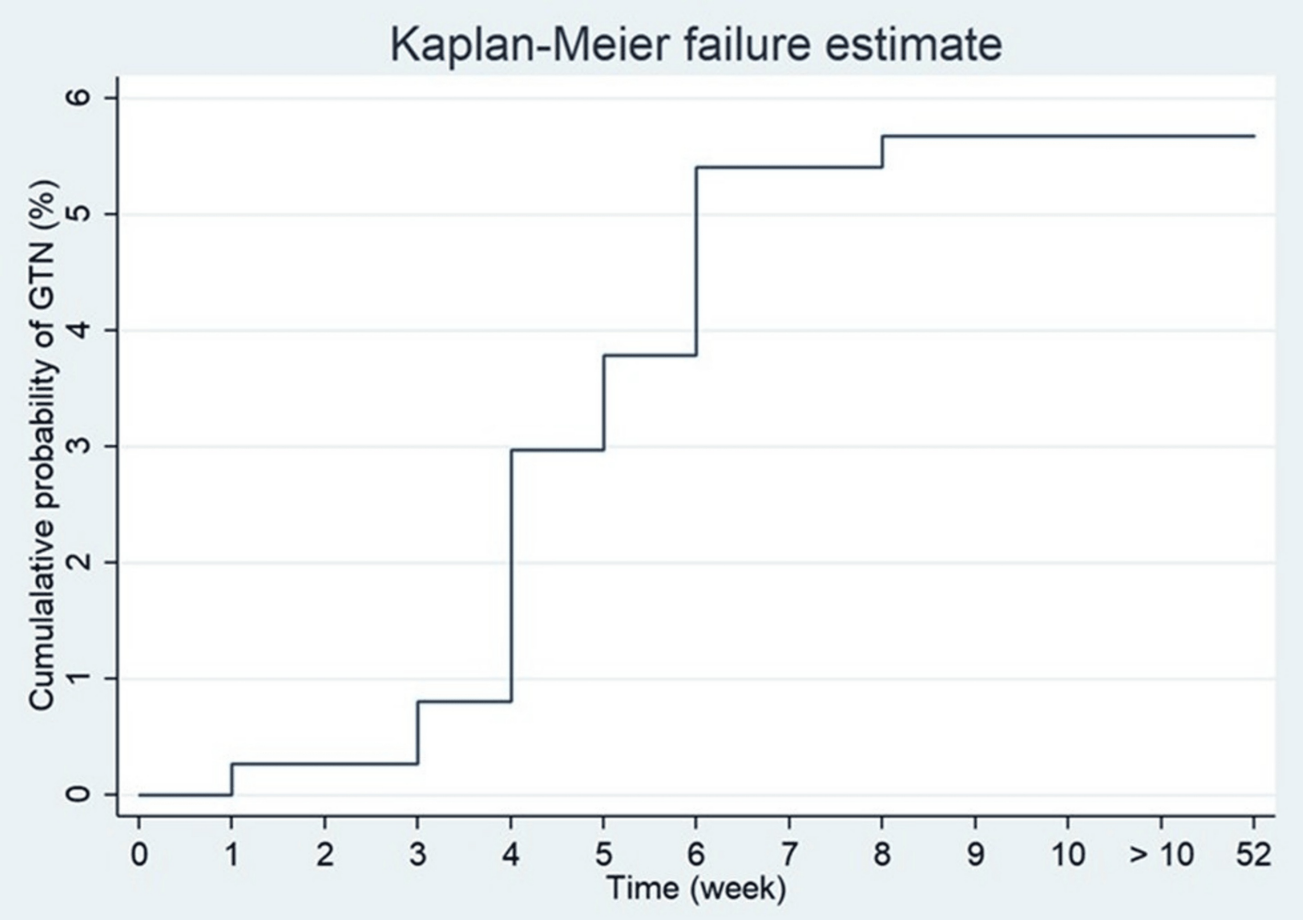
Gestational trophoblastic neoplasia rate over time (by week)

During the 1-year follow-up, the cumulative probability of GTN after 4 weeks and 8 weeks was 3.0% and 5.7%, respectively (Figure 1). The rate of detection of GTN was highest at three to six weeks after curettage abortion, peaked at week four, and no cases occurred after eight weeks of curettage (Table 2).

**Table 2:** The post-abortion cumulative rates of GTN over time (n=370) GTN: gestational trophoblastic neoplasia; CI: confidence interval

Time (weeks)	No GTN (n=370)	GTN (n=21)	Cumulative (%)	95%CI
1	370	1	0.3 %	0-0.60
2	369	0	0.3%	0-0.60
3	369	2	0.8 %	0.10-1.50
4	367	8	3.0 %	1.5-4.5
5	359	3	3.8 %	1.8-5.8
6	356	6	5.4 %	2.7-8.1
7	350	0	5.4 %	2.7-8.1
8	349	1	5.7 %	2.7-8.7
9	0	0	5,7 %	2.7-8.7
52	0	0	5,7 %	2.7-8.7

To identify factors related to GTN in patients with PHM after curettage termination, the first 21 pairs of univariates were analyzed. To control confounders and co-factors, GTN-related factors with p<0.25 in the Cox multivariable regression model were selected. This included the following six factors: age, previous history of childbirth, previous history of miscarriage or abortion, presence of vaginal bleeding, β-hCG levels at the time of diagnosis, and a WHO 1983 risk classification of ‘low’ for HM.

In the Cox multivariable regression model, only one factor related to GTN was recorded: a history of miscarriage/termination (Table 3). PHM patients with this history demonstrated a 2.84-fold increased risk of developing GTN compared with those without (hazard ratio (HR=2,84 95%CI: 1.05-7.69). The remaining five aforementioned factors exhibited no statistical significance in the risk of progressing to GTN.

**Table 3:** GTN-correlated factors (n=370) GTN: gestational trophoblastic neoplasia; CI: confidence interval; HR: hazard ratio; β-hCG: beta-human chorionic gonadotropin; *Univariable Cox regression model; **Multivariable Cox regression model

Factors	Univariate/GTN HR (95%CI)	p-value*	Multivariate/GTN HR (95%CI)	p-value**
Age (years)				
<30	1		1	
30-40	2.9(0.81-10.4)	0.10	3.04(0.71-13.1)	0.14
>40	4.87(1.26-18.8)	0.20	2.91(0.55-15.5)	0.21
Number of births				
Not yet	1		1	
1 time	1.83(0.60-5.60)	0.29	1.57(0.40-6.17)	0.52
≥2 time	2.38(0.78-7.28)	0.13	1.47(0.50-4.37)	0.49
History of miscarriage or termination				
No	1		1	
Yes	3.20(1.24-8.23)	0.016	2.84(1.05-7.69)	0.04
Vaginal bleeding				
No	1		1	
Yes	1.88(0.80-4.44)	0.15	1.43(0.58-3.56)	0.44
β-hCG at the time of diagnosis (mUI/mL)				
<100.000	1		1	
≥100.000	3.17(1.07-9.42)	0.04	2.64(0.82-8.51)	0.11
Risk by WHO 1983 classification				
Low	1		1	
High	3.79(1.57-9.14)	0.003	2.57(0.78-8.54)	0.12

## Discussion

The cumulative rate of progression to GTN in this study was 21/370, equivalent to 5.7%, equivalent to other studies on PHM patients, specifically, the rate of trophoblast proliferation in Lavie et al. [7] was 4.0% and Feltmate et al. [5] was 5.6%. This rate is considerably lower when compared with studies both in Vietnam and globally in a group of HM patients in general. Specifically, the rate of progression to GTN in Tuan et al. [12] conducted at Tu Du Hospital was 17.3%, approximately 3 times higher than the present study, while Hang et al. [13] also showed an incidence of 19.5%, which is 3.4 times higher. A multicenter study by Bakhtiyari et al. [14] gave an incidence of 18.6% in the high-risk group and 13.3% in the low-risk group, 3.3 times and 2.3 times higher, respectively, than the present study. These results suggest that the rate of progression to GTN in patients with PHM may be between 4-6%, which is approximately 3 times lower than in patients with HM in general.

After multivariable analysis, only one factor demonstrating a link between GTN in PHM patients was identified: a previous history of miscarriage or termination. Data indicate that a history of miscarriage or termination was a 2.84-fold increased risk of progression to GTN compared with patients without this history. In Messerli's multi-center case-control study of 190 cases of GTN and 189 control cases [15], the results showed that a history of miscarriage had an odds ratio of 2.32 (p=0.02). Parazzini et al.'s study of 49 PHM patients and over 139 CHM patients, showed that PHM patients with a history of miscarriage had a 2-fold increased GTN risk [16]. Further studies on HM patients also demonstrate a history of miscarriage/termination as a factor relating to GTN with a 2.53 times greater risk, according to Bakhtiyari et al. [14], and 1.6 times greater risk according to Hang [13]; consequently, a history of miscarriage/termination is evidenced as a risk factor for progression to GTN, not only in PHM patients but in HM patients in general. Messerli et al. [15] suggested that patients with PHM, which progressed to GTN had certain genetic characteristics like those of patients with a history of miscarriage. Baltazar also hypothesized that choriocarcinoma may originate from a defective gene that produces pathological oocytes [17].

In conclusion, the present study found that the GTN incidence in patients with PHM in a southern Vietnamese hospital was 5.7%. The history of miscarriage or termination was shown to be the risk factor most strongly associated with GTN.

Clinical implications

In clinical practice, it may be necessary to advise patients on the risks of developing GTN after curettage termination in high-risk PHM, especially those with an existing history of miscarriage or termination. It should also be noted that PHM is not completely benign. Ongoing management of patients following PHM should occur from the diagnosis of GTN throughout treatment and be closely followed up.

Limitations

This is a retrospective cohort study; therefore, errors related to record retrievals, such as missing information, missing records, and errors, in the original records are unavoidable. Furthermore, there are some subjective variables, such as the last menstrual period, abdominal pain symptoms, nausea, and fatigue; or subjective variables in clinical practice such as uterine size. All the above limitations may affect the results of this study. The merit of this article is to give the data on PHM in Vietnam in one hospital. This paper may be useful for the Vietnamese population but may not be suitable for the worldwide population. No new intervention was performed.

## Conclusions

The rate of GTN in PHM patients was 5.7%. Patients who had a history of miscarriage or termination were 2.84 times more likely to develop GTN than patients who did not. Previous literature suggested that PHM is a benign form of HM, as the gross pathology, pregnancy progression, and β-HCG levels differ from those of CHM. The present study is one of few studies on patients with PHM alone, which has shown that this is a disease that has malignant potential to GTN. Therefore, the study contributes to refuting the above point of view and provides more information for clinical practitioners in counseling and managing PHM.
